# Supplementary material for: The Dual Prey-Inactivation Strategy of Spiders—In-Depth Venomic Analysis of Cupiennius salei
Source: Toxins (Basel). 2019 Mar 19;11(3):167. doi: 10.3390/toxins11030167 (PMC6468893; doi:10.3390/toxins11030167)
Supplement: Supplementary file 1 [file toxins-11-00167-s001.zip › Supplementary Fig S2.1-S2.22/Supplementary information S2.1 _S2.21 Fig.pdf]

Supplementary Fig S2.1-S2.21

Supplementary Fig S2.1

1. CsTx-1a,b

CLUSTAL O (1.2.4) multiple sequence alignment of CsTx-1a,b from *Cupiennius salei* (CUPSA) with peptides from *Nephila pilipes* (NEPPI), *Viridasius fasciatus* (VIRFA), and *Lycosa singoriensis* (LYCSI)

```
Identifier                      mature peptide sequence                                                                family
NEPPI_DN30656_c0_g6_i1_5        -HEEKACIEKHHECTHDKENCCEGKYFQYKCKCYDVTNEEGNQVQRCACKNPFKYKAAEFFFSIGKKL-------------   67  SN_19_13
sp|P81694|TXC1_CUPSA            -----SCIPKHEECTNDKHNCCRKGLFKLKCQCSTFDDESGQPTERCACGRPMGHQAIETGLNIFRGLFKGKKKNKKTK(G)  75  SN_19_13
VIRFA_DN65866_c0_g1_i1_4        ESEEKRCIPKHYECTHHKDECCKGHLFHYKCRCYKIAGDKGEESKRCACVTPLHHKLLELVSMVAQNIIK(G)--------   71  SN_19_13
sp|B6DCP0|TX151_LYCSI           ----KDCIPKHHECTSNKHGCCRGHLFKYKCQCTTVVTQSGEETERCFCGTPPHHKAAELVVGFGKKIF(G)---------   66  SN_19_03
                                      ** ** *** .*. **.   *: **:*  .  :.*:  :** *  *  ::  *    . : :
```

Percent Identity Matrix - created by Clustal2.1

Nr.	1	2	3	4
2: NEPPI_DN30656_c0_g6_i1_5	100.00	42.86	43.28	48.44
3: sp|P81694|CsTx-1_CUPSA	42.86	100.00	39.39	52.31
4: VIRFA_DN65866_c0_g1_i1_4	43.28	39.39	100.00	53.03
5: sp|B6DCP0|TX151_LYCSI	48.44	52.31	53.03	100.00

Supplementary Fig S2.2

2. CsTx-10a/CsTx-9a,b and CsTx-1a,b

CLUSTAL O (1.2.4) multiple sequence alignment of CsTx-1a,b, CsTx-10a, and CsTx-9a from *Cupiennius salei* (CUPSA) with *Latrodectus mactans* (LATMA) and δ-miturgitoxin-Cp2a from *Cheiracanthium punctorium* (CHEPU)

```
Identifier                          mature peptide sequence                                                                       family
tr|A0A1V0FWS6|A0A1V0FWS6_LATMA      ---DECTPLTHDCTHDRHSCCRGPTFKYKCDCLYPFDNSTSAWDQTELCFCVEPGVHHFLDEVMDKTIGIF(G)----------      69  SN_19_00
sp|A0A059T2H4|TX2A_CHEPU_Nterm      --GKKCIERNKECTNDRHGCCRGKIFKDKCECVGSG--------GKERCVCKQKKWAKIIESYIGDIPTLPKPE--------       64  SN_19_00 (SN_47_01)
sp|A0A059T2H4|TX2A_CHEPU_Cterm      --DDKCVPKHEDCSERKNDCCKSGLFTLKCKCYDMQDDED--GKKTELCGCVQPFEHKAIEQALRFGKWMV(G)---------       68  SN_19_13 (SN_47_01)
sp|B3EWT0|TXC10_CUPSA               KDKENCIGKHHECTDDRDNCCKGKLFRYQCQCFKVID-GK--K-ETKRCACVTPLHYKMAEMAVSVFKKMFKN---------       69  SN_19_14
sp|P81694|TXC1_CUPSA                ----SCIPKHEECTNDKHNCCRKGLFKLKCQCSTFDD-ES--GQPTERCACGRPMGHQAIETGLNIFRGLFKGKKKNKKTK(G)      75  SN_19_13
sp|P58604|TXC9_CUPSA                KDDKNCIPKHHECTNDKKNCCKKGLTKMKCKCFTVAD-AK--GATSERCACDSSLLQKFGFTGLHIIKGLF-----------       68  SN_19_13
                                        .*    .:*:. :..**:      :*.*             .: * *      :     :     :
```

Percent Identity Matrix - created by Clustal2.1

Nr.	1	2	3	4	5	6
1: tr|A0A1V0FWS6|A0A1V0FWS6_LATMA	100.00	29.51	31.34	30.77	35.38	29.23
2: sp|A0A059T2H4|TX2A_CHEPU_Nterm	29.51	100.00	25.81	31.75	38.71	34.43
3: sp|A0A059T2H4|TX2A_CHEPU_Cterm	31.34	25.81	100.00	33.33	44.62	36.36
4: sp|B3EWT0|TXC10_CUPSA	30.77	31.75	33.33	100.00	41.54	46.27

5: sp|P81694|TXC1_CUPSA	35.38	38.71	44.62	41.54	100.00	56.25
6: sp|P58604|TXC9_CUPSA	29.23	34.43	36.36	46.27	56.25	100.00

G C-terminal amidation, proposed α-helical part (GOR4 prediction)
Combet C., Blanchet C., Geourjon C. and Deléage G., TIBS 2000 March Vol. 25, No 3 [291]:147-150

Supplementary Fig S2.3

3. CsTx-23a,b

CLUSTAL O (1.2.4) multiple sequence alignment of CsTx-23a,b from *Cupiennius salei* (CUPSA) with putative neurotoxins from *Lycosa singoriensis* (LYCSI), *Viridasius fasciatus* (VIRFA), *Heteropoda venatoria* (HETVE), and *Heteropoda davidbowie* (HETDA)

```
Identifier                            mature peptide sequence                                  family
tr|A0A1V0FWV6|A0A1V0FWV6_VIRFA    --QCIRYEHVCSFNKGECCTGLKCECYDRYIKGEKGEEKCWCIEKDVMYKKRGE---     52  SN_19_06
sp|B6DCV8|TX608_LYCSI             --ACIPLEKECTKTPGNCCSGLKCDCYRRFEQGVAKGVQCWCIEKDVTYKGV-----     50  SN_19_06
sp|B6DCV2|TX602_LYCSI             --ACIPLEKECTKTPGNCCSGLKCNCYRRFEQGVAKGIQCWCIEKDVTYKGV-----     50  SN_19_06
sp|B6DCW0|TX610_LYCSI             --ACIPLEKECTKTPGNCCSGLKCDCYRRFEQGVAKGIQCWCIEKDVTYKGI-----     50  SN_19_06
CsTx-23a_CUPSA                    --ACIEYELECTENKDNCCSDYKCQCYRRFVKGVDKGPSCFCLEKDVKYKPL-----     50  SN_19_06
CsTx-23b_CUPSA                    --ACIGFELECTKDKKNCCSDYKCQCYRRFVKGVDKGQKCWCLDKDVKYKPL-----     50  SN_19_06
tr|A0A1V0FWV4|A0A1V0FWV4_HETDA    --QCTPQEHRCFKGAPKCCGGFDCQCYTPIVNGVKEEPTCWCNEPNVIYEYAFKAQY     55  SN_19_00
tr|A0A1V0FWQ9|A0A1V0FWQ9_HETDA    --QCIKLEGECTKNKDNCCAEHRCRCYDKYVNGIKTEVRCWCFEKDVTYKPTFEIK-     54  SN_19_06
                                     *      *      **    * **    :       * *
```

Percent Identity Matrix - created by Clustal2.1

Nr.	1	2	3	4	5	6	7	8
1: tr|A0A1V0FWV6|A0A1V0FWV6_VIRFA	100.00	50.00	50.00	50.00	48.00	46.00	38.46	51.92
2: sp|B6DCV8|TX608_LYCSI	50.00	100.00	96.00	96.00	60.00	62.00	38.00	52.00
3: sp|B6DCV2|TX602_LYCSI	50.00	96.00	100.00	96.00	60.00	62.00	38.00	50.00
4: sp|B6DCW0|TX610_LYCSI	50.00	96.00	96.00	100.00	60.00	62.00	38.00	50.00
5: CsTx-23a_CUPSA	48.00	60.00	60.00	60.00	100.00	82.00	36.00	52.00
6: CsTx-23b_CUPSA	46.00	62.00	62.00	62.00	82.00	100.00	36.00	50.00
7: tr|A0A1V0FWV4|A0A1V0FWV4_HETDA	38.46	38.00	38.00	38.00	36.00	36.00	100.00	40.74
8: tr|A0A1V0FWQ9|A0A1V0FWQ9_HETDA	51.92	52.00	50.00	50.00	52.00	50.00	40.74	100.00

Supplementary Fig S2.4

4. CsTx-36a,b,c & CsTx-28a,b

CLUSTAL O (1.2.4) multiple sequence alignment of CsTx-36a,b,c and CsTx-28a,b from *Cupiennius salei* (CUPSA) with (putative) neurotoxins from *Viridasius fasciatus* (VIRFA), *Lycosa singoriensis* (LYCSI), and *Phoneutria reidyi* (PHORE)

```
Identifier                            mature peptide sequence                               family
tr|A0A1V0FWW7|A0A1V0FWW7_VIRFA    APKCIDVYETCN-KGIPCCEDRPCRCNIVMDNCICKKTIA----ELFGGK         45  SN_02_10
```

```
CsTx-28a                            --ECIQPYKTCSSGGTRCCGRASCRCPISGSNCKCTKPVSQIIADWFS(G)    47  SN_02_03
CsTx-28b                            --ECIEPYKTCSWSGTRCCGRASCRCSIAGTNCKCTKPASQIIADWFS(G)    47  SN_02_03
sp|B6DD53|TXG02_LYCSI               --SCAKEYQWCDWNTRPCCDNISCICSWIGTNCECKKGIICTIKDWYKGK     48  SN_02_04
sp|P84000|TXC5_PHORI                --SCADAYKSCD--SLKCCNNRTCMCSMIGTNCTCRKK------------     34  SN_02_13
CsTx-36aS1_1                        --SCAEAYQTCD--SIPCCNERSCVCNWLGKECKCKKSLGELIDTLLGS-     45  SN_02_04
                                      .* . *: *.     **    * *     :* * *
```

Percent Identity Matrix – created by Clustal2.1

Nr.	1	2	3	4	5	6
1: tr|A0A1V0FWW7|A0A1V0FWW7_VIRFA	100.00	42.86	42.86	37.21	38.24	43.90
2: CsTx-28a_CUPSA	42.86	100.00	85.11	36.17	38.24	33.33
3: CsTx-28b_CUPSA	42.86	85.11	100.00	42.55	44.12	35.56
4: sp|B6DD53|TXG02_LYCSI	37.21	36.17	42.55	100.00	55.88	44.44
5: sp|P84000|TXC5_PHORE	38.24	38.24	44.12	55.88	100.00	52.94
6: CsTx-36a,b,c_CUPSA	43.90	33.33	35.56	44.44	52.94	100.00

Supplementary Fig S2.5

5. CsTx-17 & CsTx-25

CLUSTAL O (1.2.4) multiple sequence alignment of CsTx-17 and CsTx-25 from *C. salei* (CUPSA) with (putative) neurotoxins from *Agelenopsis aperta (AGEAP)*, *V. fasciatus* (VIRFA), and the pseudoscorpion *Synsphyronus apimelus* (Sapi)

```
Identifier                          mature peptide sequence                                family
Sapi_DN107241_c0_g1_i1              --ACI-RRGGTCDHRPNDCCYSSSCRCNLWGTNCRCQRAGLFQRWG-------    43  SN_02_06
Sapi_DN110686_c0_g1_i1              --SCI-RRGGMCDHRPHDCCYSASCRCNLWGTNCRCQRPGFLQSLGR------    44  SN_02_06
tr|A0A1V0FWP3|A0A1V0FWP3_VIRFA      -GQC-ADVWKRCG-NGVECCCNRSCICNVTRTKCKCHRFH-EVSHGYTAECLV    49  SN_02_01
sp|P37045|TOG4B_AGEAP               EDNCIAEDYGKCTWGGTKCCRGRPCRCSMIGTNCECTPRLIMEGLSFA-----    48  SN_02_03
sp|P30288|TOG4A_AGEAP               KKKCIAKDYGRCKWGGTPCCRGRGCICSIMGTNCECKPRLIMEGLGLA-----    48  SN_02_03
sp|B3EWT2|TXC17_CUPSA               --GCI-PKHKRCTWSGPKCCNNISCHCNISGTLCKCRPGLFGW----------    40  SN_02_03
CsTx-25_CUPSA                       --YCI-PKWRRCTWGGPKCCAGRSCDCNVTRTNCRCSPRLFGL(G)-------    41  SN_02_03
                                       *       *      ** .  * *.:  * *.*
```

Percent Identity Matrix – created by Clustal2.1

Nr.	1	2	3	4	5	6	7
1: Sapi_DN107241_c0_g1_i1	100.00	79.07	29.27	32.56	32.56	32.50	34.15
2: Sapi_DN110686_c0_g1_i1	79.07	100.00	30.95	34.09	34.09	35.00	36.59
3: tr|A0A1V0FWP3|A0A1V0FWP3_VIRFA	29.27	30.95	100.00	25.00	31.82	42.11	43.59
4: sp|P37045|TOG4B_AGEAP	32.56	34.09	25.00	100.00	70.83	42.50	53.66
5: sp|P30288|TOG4A_AGEAP	32.56	34.09	31.82	70.83	100.00	42.50	51.22
6: sp|B3EWT2|TXC17_CUPSA	32.50	35.00	42.11	42.50	42.50	100.00	60.00
7: CsTx-25_CUPSA	34.15	36.59	43.59	53.66	51.22	60.00	100.00

Supplementary Fig S2.6

6. CsTx-34a,b

CLUSTAL O (1.2.4) multiple sequence alignment of CsTx-34a,b from *Cupiennius salei* (CUPSA) with (putative) neurotoxins from *Phoneutria nigriventer* (PHONI), *Dolomedes mizhoanus* (DOLMI), and *Alopecosa marikovskyi* (ALOMA), formerly classified as *Geolycosa sp* (GEOA2)

```
Identifier                     mature peptide sequence                      family
sp|P81791|TX35_PHONI        --GCIGRNESCKFD-RHGCCWPWSCSCWNKEGQPES---DVWCECSLKIGK----   45  SN_02_16
CsTx-34a,b_CUPSA            -KDCIKRDHSCTHD-KNGCCWPQYCDCWSNRRT-----YEYKCECRRRHLLFLQV   48  SN_02_16
tr|S5N3V5|S5N3V5_DOLMI      -KDCIKWRLSCGGEDDNSCCWPYGCQCWSQTVSKSSSRKVRKCQCRLW-------   47  SN_29_02
sp|A9XDG3|TLCOG_GEOA2_ALOMA EKSCITWRNSCMHY-DKGCCFPWTCVCWSQTVSRNSSRKEKKCQCRLR-------   47  SN_29_01
                              .*     **     :.**:*  * **.:            *:*
```

Percent Identity Matrix - created by Clustal2.1

Nr.	1	2	3	4
1: sp|P81791|TX35_PHONI	100.00	40.48	35.71	38.10
2: CsTx-34a,b_CUPSA	40.48	100.00	48.78	48.78
3: tr|S5N3V5|S5N3V5_DOLMI	35.71	48.78	100.00	63.04
4: sp|A9XDG3|TLCOG_GEOA2_ALOMA	38.10	48.78	63.04	100.00

Supplementary Fig S2.7

7. CsTx-25 & agatoxin-like peptides

CLUSTAL O (1.2.4) multiple sequence alignment of CsTx-25 from *C. salei* (CUPSA) with (putative) neurotoxins from *Agelenopsis orientalis* (AGEOR), the remipide *Xibalbanus tulumensis* (SPETU), the pseudoscorpion *Synsphyronus apimelus* (Sapi), agatoxin-like peptide from the hemocytes of *C. salei* (agatoxin-like CUPSA_Hemo), agatoxin-like peptide from the neuroendocrine system of *Apis mellifera* (APIME), agatoxin-like peptide from the salivary glands of the tick *Ixodes ricinus* (IXORI), and the (putative) neurotoxins CsTx-28a and 31a from *C. salei.*

```
Identifier                       mature peptide sequence                                  family
sp|Q5Y4U4|TX20A_AGEOR            SEDQCIGRSCTCDTSSTSCCPYAACRCNLWKTSCKCQRTGRK--WATPCKEIYSPN 54  SN_02_06
contig00124_Remipedia_XIBTU      --LFCIRHHKYCGHRPNRCCSGLFCRCNTFGTNCRCQSKGAL--GKLISGK----- 47  SN_02_06
Sapi_DN110686_c0_g1_i1           ---SCIRRGGMCDHRPHDCCYSASCRCNLWGTNCRCQRPGFL--QSLGR------- 44  SN_02_06
tr|A0A088AGX1_APIME              ---ACIPRSGNCDHRPKDCCYSSSCRCNLWGSNCRCQRMGLF--QKWG-------- 43  SN_02_06
agatoxin-like CUPSA_Hemo         ---SCIRRGSSCDHRPSDCCFNSSCRCNLWGTNCRCQRAGLF--QKWG-------- 43  SN_02_06
tr|A0A147BFN0_IXORI              ---SCIRRGGSCDHRPNDCCYNSSCRCNLWGTNCRCQRMGLF--QKWGKK------ 45  SN_02_06
CsTx-28a_CUPSA                   ---ECIQPYKTCSSGGTRCCGRASCRCPISGSNCKCTKPVSQIIADWFSG------ 47  SN_02_03
CsTx-25_CUPSA                    ---YCIPKWRRCTWGGPKCCAGRSCDCNVTRTNCRCSPRLFGLG------------ 41  SN_02_03
CsTx-31a_CUPSA                   ---GCVQAGKPCTWGKTKCCGGIICKCNYSKTKCHCKPPSLDFGLG---------- 43  SN_02_03
                                     *:     *      **    * *    :.*.*
```

Percent Identity Matrix – created by Clustal2.1

Nr.	1	2	3	4	5	6	7	8	9
1: sp|Q5Y4U4|TX20A_AGEOR	100.00	29.79	45.45	44.19	44.19	44.44	31.11	28.21	29.27
2: contig00124_Remipedia_XIBTU	29.79	100.00	52.27	46.51	51.16	51.11	35.56	35.90	29.27
3: Sapi_DN110686_c0_g1_i1	45.45	52.27	100.00	72.09	74.42	77.27	34.09	38.46	29.27
4: tr|A0A088AGX1_APIME	44.19	46.51	72.09	100.00	76.74	83.72	34.88	35.90	24.39
5: agatoxin-like CUPSA_Hemo	44.19	51.16	74.42	76.74	100.00	90.70	32.56	35.90	29.27
6: tr|A0A147BFN0_IXORI	44.44	51.11	77.27	83.72	90.70	100.00	31.11	35.90	29.27
7: CsTx-28a_CUPSA	31.11	35.56	34.09	34.88	32.56	31.11	100.00	31.71	34.88
8: CsTx-25_CUPSA	28.21	35.90	38.46	35.90	35.90	35.90	31.71	100.00	41.46
9: CsTx-31a_CUPSA	29.27	29.27	29.27	24.39	29.27	29.27	34.88	41.46	100.00

Supplementary Fig S2.8

8. CsTx-41 & CsTx-42a,b,c

CLUSTAL O (1.2.4) multiple sequence alignment of CsTx-41 & CsTx-42a,b,c from *Cupiennius salei* (CUPSA) with (putative) neurotoxins from *Phoneutria nigriventer* (PHONI), *Phoneutria keyserlingi* (PHOKE), and *Dolomedes fimbriatus* (DOLFI)

```
Identifier                               mature peptide sequence                                                        family
CsTx-41_CUPSA                       -----AACTDLGGECQ---TDCDCCRRNGYCSCSWL---FNHCTCQPGSNANSGSICHRKNRECGL-------RMRCPKNCTTKKCM(G)K-71   SN_04_04
CsTx-42b_CUPSA                      ------VCVKPKESCG---NDCQCCVDNEYCYCAFI---WWSCTCRPSDNSNRWSVCMKKNRECGL-------NMTCPKNCTTKRCM(G)K-70   SN_04_04
CsTx-42a_CUPSA                      ------VCVKPKESCD---NDCQCCVANEYCYCAFG---WWSCTCRPSDNSNRWGVCMKKNRECGL-------NMTCPKNCTTKRCM(G)K-70   SN_04_04
CsTx-42c_CUPSA                      ------VCVKPKESCD---NDCQCCVANEYCYCAFG---WWSCTCRPSDNSNRWGVCMKKNRECGL-------NMTCQKNCTTKRCM(G)K-70   SN_04_04
sp|P84062|TX1A_PHOKE                --AELKSCFPVGHECDDDASNCNCCGDDVYCACGWG---RWNCKCKVADQSYAYGICKDKVN-CPNRHLWPA--KECKMPCRRN-CG----  78   SN_04_02
sp|P17727|TXL1_PHONI                --AELTSCFPVGHECDGDASNCNCCGDDVYCGCGWG---RWNCKCKVADQSYAYGICKDKVN-CPNRHLWPA--KVCKKPCRRN-CGG---  79   SN_04_02
tr|A0A0K1D8V8|A0A0K1D8V8_DOLFI      EVTRNTPCLKLGEKCVKGESDCQCCRDNAVCSCSWL--IWSHCTCQVFGASESYSTCLKKVN-CPNRYQWGSGSKTCTKPCLRKHCRGK--  86   SN_04_05
sp|P81790|TX34_PHONI                ------SCINVGDFCDGKKDDCQCCRDNAFCSCSVIFGYKTNCRCEVGTTATSYGICMAKHK-CGR-------QTTCTKPCLSKRCKKNHG  77   SN_04_01
sp|P84093|TOPC5_PHONI               -----GNCIELNNDCDGSKDDCQCCRDNAYCSCYNFFGIKSGCKCSVGNSGTGYSVCLKKLE-CPNRRAWTSWKKECTKPCIGKRC-----  80   SN_04_03
                                           *      *     :*:**  :  * *         * *     .   . *  * . *            *   *  : *
```

Post-translational removement

Percent Identity Matrix - created by Clustal2.1

Nr.	1	2	3	4	5	6	7	8	9
1: CsTx-41_CUPSA	100.00	54.29	52.86	51.43	30.30	32.84	47.14	40.58	37.31
2: CsTx-42b_CUPSA	54.29	100.00	94.29	92.86	30.77	33.33	44.93	37.68	39.39
3: CsTx-42a_CUPSA	52.86	94.29	100.00	98.57	33.85	36.36	42.03	37.68	37.88
4: CsTx-42c_CUPSA	51.43	92.86	98.57	100.00	33.85	36.36	42.03	37.68	37.88
5: sp|P84062|TX1A_PHOKE	30.30	30.77	33.85	33.85	100.00	93.59	39.74	38.24	37.84
6: sp|P17727|TXL1_PHONI	32.84	33.33	36.36	36.36	93.59	100.00	43.04	40.58	39.19
7: tr|A0A0K1D8V8|A0A0K1D8V8_DOLFI	47.14	44.93	42.03	42.03	39.74	43.04	100.00	45.21	51.28
8: sp|P81790|TX34_PHONI	40.58	37.68	37.68	37.68	38.24	40.58	45.21	100.00	54.17
9: sp|P84093|TOPC5_PHONI	37.31	39.39	37.88	37.88	37.84	39.19	51.28	54.17	100.00

Supplementary Fig S2.9

9. CsTx-37

CLUSTAL O (1.2.4) multiple sequence alignment of CsTx-37 from *Cupiennius salei* (CUPSA) with (putative) neurotoxins from *Lycosa singoriensis* (LYCSI), *Heteropoda venatoria* (HETVE), *Agelena orientalis* (AGEOR), *Dolomedes fimbriatus* (DOLFI), and *Dolomedes sulfureus* (DOLSU)

```
Identifier                          mature peptide sequence                                                             family
sp|B6DD27|TXD09_LYCSI               SCADMGQDCK--DDCDCCLNIATCNCWFG-RYFCSC-TFGDYQTCLRKKGKCKRNRPQSCPRSNLNRKKG----------------- 66  SN_05_05
tr|A0A088BPD5|A0A088BPD5_HETVE      SCSPPGDKCT--SDCDCCISYVKCDCESGD---CVCKESSDASVCERKKKMCLNPAAFNFPDGPCLVKSRKKRIGENFNENNTANIA 82  SN_05_07
sp|Q5Y4U3|TXAG9_AGEOR               ACIKEGEKCA--GDCQCCGKWSYCSCPLFGALGCSC-IIGDAMVCVRKKKECRTSDVMNTPPGGCFSSSKRRHGR------------ 72  SN_05_11
tr|A0A0K1D8C2|A0A0K1D8C2_DOLFI      SCTPLGGDCEPGNDCKCCNKWAYCKCTLFNKWGCSC-VFGDAMVCQRKKEQCSDPQVMDSPKGPCFYSTRKNKRRMTG--------- 77  SN_05_07
tr|A0A0P0DIM7|A0A0P0DIM7_DOLSU      SCVALGGECN-GNDCKCCNKWTYCKCPLWGVLGCSC-VFGDSMVCVRKRESCKNPEVMDYPKGSCFSSKRGNNRRGR---------- 75  SN_05_07
CsTx-37_CUPSA                       ACVPLGEECN-GNDCKCCNKWTYCKCPFGTGFACSC-VFGGAMVCERKKKKCKNPEVMNTPPGPCFSGR(G)--------------- 68  SN_05_07
                                     :*   * .*   .**.** .   *.*        .  - ..  .* **:  *      . *
```

Percent Identity Matrix - created by Clustal2.1

Nr.	1	2	3	4	5	6
1: sp|B6DD27|TXD09_LYCSI	100.00	31.25	30.30	36.36	34.85	37.88
2: tr|A0A088BPD5|A0A088BPD5_HETVE	31.25	100.00	34.29	35.62	30.56	41.54
3: sp|Q5Y4U3|TXAG9_AGEOR	30.30	34.29	100.00	50.00	52.78	56.72
4: tr|A0A0K1D8C2|A0A0K1D8C2_9ARAC	36.36	35.62	50.00	100.00	66.67	60.29
5: tr|A0A0P0DIM7|A0A0P0DIM7_9ARAC	34.85	30.56	52.78	66.67	100.00	67.65
6: CsTx-37_CUPSA	37.88	41.54	56.72	60.29	67.65	100.00

Supplementary Fig S2.10

10. CsTx-38a,b,c

CLUSTAL O (1.2.4) multiple sequence alignment of CsTx-38a,b,c from *C. salei* (CUPSA) with (putative) neurotoxins from *Ancylometes rufus* (ANCRU), *Oxyopes heterophthalmus* (OXYHE), *Dolomedes sulfureus* (DOLSU), and *Lycosa singoriensis* (LYCSI)

```
Identifier                          mature peptide sequence                                                             family
ANCRU_DN33832_c1_g5_i3_4            -ACVWNGNSCNNDCDCCGDINHCSCTL----GLFACSCTIGYDSTCWDKQEKCTGETRPKKCQASFKGRNHG-------------   67  SN_05_04
OXYHE_DN44064_c2_g5_i1_4            -ECVFPGGQCKDDCDCCVANNYCHCPFWGIS-NKLCSCIFGDYQVCESRRKKCTR-NRPQVCRWPPRRRNSGG------------   70  SN_05_01
tr|A0A0P0CV52|A0A0P0CV52_DOLSU      -SCVSPGQVCKDDCDCCVNNNYCYCPLWGILGADSCSCIFGDDFVCRERMGKCSR-NRPQKCPTSRSTRRRG-------------   70  SN_05_01
sp|B6DD24|TXD06_LYCSI               -SCAGMGRDCKDDCDCCLNIATCNCWF----GRYFCSCTFGDYQTCLRKKGKCKR-NRPQSCPRSNLNRKKG-------------   66  SN_05_05
CsTx-38c_CUPSA                      GCEYNMGKECKDDCDCCVNISKCNCML----GSYLCSCGYGDRYTCLDKVRKCTK-NRPHECPLPDLLPTRGKGPNYSNIVNRLP   80  SN_05_08
CsTx-38a_CUPSA                      GCEYNMGKECKDDCDCCVNISKCNCML----GSYLCSCGYGNRYTCLDKVRKCTK-NRPHECPLPDLLPTRGKGPNYGNIMNRLP   80  SN_05_08
CsTx-38b_CUPSA                      GCEYNMGKECKDDCDCCVNISKCNCML----GSYLCSCGYGDRYTCLDKVRKCTK-NRPHECPLPDLLPTRGKGPNYGNIMNRLP   80  SN_05_08
                                          *  *::*****     * * :        ***  *   .*  :  **.  .**: *
```

Percent Identity Matrix - created by Clustal2.1

Nr.	1	2	3	4	5	6	7
1: ANCRU_DN33832_c1_g5_i3_4	100.00	40.00	43.94	42.42	40.91	40.91	40.91

2: OXYHE_DN44064_c2_g5_i1_4	40.00	100.00	60.87	49.23	41.79	40.30	41.79
3: tr|A0A0P0CV52|A0A0P0CV52_DOLSU	43.94	60.87	100.00	53.03	45.45	43.94	45.45
4: sp|B6DD24|TXD06_LYCSI	42.42	49.23	53.03	100.00	53.03	51.52	53.03
5: CsTx-38c_CUPSA	40.91	41.79	45.45	53.03	100.00	96.25	97.50
6: CsTx-38a_CUPSA	40.91	40.30	43.94	51.52	96.25	100.00	98.75
7: CsTx-38b_CUPSA	40.91	41.79	45.45	53.03	97.50	98.75	100.00

Supplementary Fig S2.11

11. CsTx-24a,b & CsTx-39

CLUSTAL O (1.2.4) multiple sequence alignment of CsTx-24a,b & CsTx-39 from *C. salei* (CUPSA) with (putative) neurotoxins from *Lycosa singoriensis* (LYCSI), *Stegodyphus mimosarum* (STEMI), *Eresus cinnaberinus* (ERICI), *Viridasius fasciatus* (VIRFA), and *Dolomedes mizhoanus* (DOLMI)

```
Identifier                          mature peptide sequence                                family
sp|B6DD18|TXC06_LYCSI               GCADLRQPCTKGDDCSCCGSDGVCNCEHLHKTG-CFCKTAGPYEKLKKWFKRCPKY-   55  SN_31_05
tr|A0A087UMF8|A0A087UMF8_STEMI      ACVGTKEICTEGDDCSCCGEWDMCDCNNPNEPNVCRCKYAMIYTRVKKMATC-----   52  SN_31_04
tr|A0A2D0PC11|A0A2D0PC11_ERECI      ACVGTKEKCVEGDDCSCCGEWDSCDCNNPNERGVCRCKYAMIYTRVKKMVKC-----   52  SN_31_04
tr|A0A087UMF7|A0A087UMF7_STEMI      ACVGTKEKCVEGDDCSCCGEWDSCDCNNPNEPNVCRCKYAMLYTRVKKMVAC-----   52  SN_31_04
tr|A0A1V0FWQ4|A0A1V0FWQ4_VIRFA      -CASQGEECADGDDCSCCGDRGKCDCNWPNKPG-CFCMQGMAYTAMKKAMICGW---   52  SN_31_04
CsTx-39_CUPSA                       SCADRKEKCTKGDDCSCCGAWNKCDCNWPDRPG-CYCMRGMMATRISKM-KCRKGKSK  56  SN_31_04
CsTx-24a_CUPSA                      ACAERKEKCTKDDDCSCCGKWDKCSCNWPGREG-CFCMRGMMATRLWKMAKC-----   51  SN_31_04
CsTx-24b_CUPSA                      ACAERKKKCTKDDDCSCCGKWDKCSCNWPGREG-CFCMRGMMATRLWKMAKC-----   51  SN_31_04
tr|S5MFF9|S5MFF9_DOLMI              ACAAFKAACTKDDDCSCCGDWDKCDCNWPGKPG-CYCMRGMIATRLKKMAIC-----   51  SN_31_04
                                     *.     *...*******  . *.*:   . . * *  .     : *
```

Post-translational removement

Percent Identity Matrix - created by Clustal2.1

Nr.	1	2	3	4	5	6	7	8	9
1: sp|B6DD18|TXC06_LYCSI	100.00	39.22	41.18	37.25	42.31	40.00	41.18	41.18	41.18
2: tr|A0A087UMF8|A0A087UMF8_STEMI	39.22	100.00	86.54	88.46	52.00	52.94	54.90	52.94	60.78
3: tr|A0A2D0PC11|A0A2D0PC11_ERECI	41.18	86.54	100.00	92.31	52.00	52.94	56.86	54.90	56.86
4: tr|A0A087UMF7|A0A087UMF7_STEMI	37.25	88.46	92.31	100.00	52.00	52.94	52.94	50.98	54.90
5: tr|A0A1V0FWQ4|A0A1V0FWQ4_VIRFA	42.31	52.00	52.00	52.00	100.00	53.85	54.00	52.00	62.00
6: CsTx-39_CUPSA	40.00	52.94	52.94	52.94	53.85	100.00	72.55	70.59	68.63
7: CsTx-24a_CUPSA	41.18	54.90	56.86	52.94	54.00	72.55	100.00	98.04	76.47
8: CsTx-24b_CUPSA	41.18	52.94	54.90	50.98	52.00	70.59	98.04	100.00	76.47
9: tr|S5MFF9|S5MFF9_DOLMI	41.18	60.78	56.86	54.90	62.00	68.63	76.47	76.47	100.00

Supplementary Fig S2.12

12. CsTx-29

CLUSTAL O (1.2.4) multiple sequence alignment of CsTx-29 from *C. salei* (CUPSA) with (putative) neurotoxins from *Dolomedes fimbriatus* (DOLFI) and *Viridasius fasciatus* (VIRFA)

```
Identifier                          mature peptide sequence                      family
tr|A0A0K1D8G8|A0A0K1D8G8_DOLFI      ENRNCGKTGDDCEYRYERCCDGFRCRYTGRTARLFICL   38  SN_34_00
CsTx-29_CUPSA                       --ENCGNVGADCEYEYERCCNDLRCRFTGRTGRLFMCL   36  SN_34_00
tr|A0A1V0FWN9|A0A1V0FWN9_VIRFA      -KENCGTTGHDCED--VKCCEGYRCLYTARTGRLWMCL   35  SN_34_00
                                   .***..* ***    :**:. ** :*.**.**::**
```

Percent Identity Matrix - created by Clustal2.1

Nr.	1	2	3
1: tr|A0A0K1D8G8|A0A0K1D8G8_DOLFI	100.00	69.44	60.00
2: CsTx-29_CUPSA	69.44	100.00	61.76
3: tr|A0A1V0FWN9|A0A1V0FWN9_VIRFA	60.00	61.76	100.00

Supplementary Fig S2.13

13. CsTx-30

CLUSTAL O (1.2.4) multiple sequence alignment of CsTx-29 from *C. salei* (CUPSA) with (putative) neurotoxins from *Dolomedes fimbriatus* (DOLFI) and *Alopecosa marikovskyi* (ALOMA), formerly classified as *Geolycosa sp* (GEOA2)

```
Identifier                          mature peptide sequence                                family
tr|A0A0K1D8H0|A0A0K1D8H0_DOLFI      --ACTKWRMSCSGENDNSCCWPYGCQCWSQTVSKKSSRKERKCQCRLW  46..SN_29_02
sp|P85079|TLCOA_GEOA2               EKSCITWRNSCMH-NDKGCCFPWSCVCWSQTVSRNSSRKEKKCQCRLW  47..SN_29_01
CsTx-30_CUPSA                       --DCVRWRRSCMG-NQNGCCLPWRCFCWSQTVSRGSTRTEQKCQCRFW  45..SN_29_01
                                       *  ** **   *::.** *: * *******: *:*.*:*****:*
```

Percent Identity Matrix - created by Clustal2.1

Nr.	1	2	3
1: tr|A0A0K1D8H0|A0A0K1D8H0_DOLDFI	100.00	66.67	62.22
2: sp|P85079|TLCOA_GEOA2	66.67	100.00	66.67
3: CsTx-30_CUPSA	62.22	66.67	100.00

Supplementary Fig S2.14

14. CsTx-26

CLUSTAL O (1.2.4) multiple sequence alignment of CsTx-26 from *C. salei* (CUPSA) with (putative) neurotoxins from *Alopecosa marikovskyi* (ALOMA), formerly classified as *Geolycosa sp* (GEOA2), *Dolomedes sulfureus* (DOLSU), *Phoneutria nigriventer* (PHONI), *Viridasius fasciatus* (VIRFA), *Agelena orientalis* (AGEOR), and *Heteropoda venatoria* (HETVE)

```
Identifier                            mature peptide sequence                            family
sp|P86269|TXPR1_GEOA2_ALOMA           GYCAEKGIRCDDIHCCTGLKCKCNASGYNCVCRKK---   35..SN_33_01
tr|A0A0P0DQC7|A0A0P0DQC7_DOLSU        GYCAENGIKCNDLHCCTGLVCKCNSSRSNCVCRKK---   35..SN_33_01
CsTx-26_CUPSA                         GYCAEKNIKCNDIHCCTGLKCKCNSSRTNCVCRKE---   35..SN_33_01
tr|A0A1V0FWV9|A0A1V0FWV9_VIRFA        GYCAEKGVKCHNIHCCENLRCKCNDDRSSCVCRKNKVS   38..SN_33_00
sp|Q5Y4U5|TXAG4_AGEOR                 GYCAEKGIKCHNIHCCSGLTCKCKG--SSCVCRK----   32..SN_33_00
tr|A0A088BP78|A0A088BP78_HETVE        GFCAEKGIKCHDLHCCTGLICKTEG--SSKVCRKK---   33..SN_33_00
                                      *:**: .::*.::*** .* *        . *
```

Percent Identity Matrix - created by Clustal2.1

Nr.	1	2	3	4	5	6
1: sp|P86269|TXPR1_GEOA2_ALOMA	100.00	77.14	80.00	62.86	71.88	66.67
2: tr|A0A0P0DQC7|A0A0P0DQC7_DOLSU	77.14	100.00	82.86	65.71	71.88	72.73
3: CsTx-26_CUPSA	80.00	82.86	100.00	65.71	71.88	63.64
4: tr|A0A1V0FWV9|A0A1V0FWV9_VIRFA	62.86	65.71	65.71	100.00	81.25	63.64
5: sp|Q5Y4U5|TXAG4_AGEOR	71.88	71.88	71.88	81.25	100.00	75.00
6: tr|A0A088BP78|A0A088BP78_HETVE	66.67	72.73	63.64	63.64	75.00	100.00

Supplementary Fig S2.15

15. CsTx-33a,b & CsTx-9

CLUSTAL O (1.2.4) multiple sequence alignment of CsTx-33a,b and CsTx-9abc from *C. salei* (CUPSA) with (putative) neurotoxins from *Cheiracanthium punctorium* (CHEPU), and *Lycosa singoriensis* (LYCSI)

```
Identifier                             mature peptide sequence                                                family
tr|A0A059T2C4|A0A059T2C4_CHEPU_C_term  PEDDKCVPKHEDCSERKNNCCKSGLFTLKCKCYDMQDDEDGKKTELCGCVQPFEHKAIEQALRFGKWMVG-------    70  SN_19_00 (SN_47_01)
tr|A0A059T2C4|A0A059T2C4_CHEPU_N_term  --GKKCIERNKECTNDRHGCCRGKIFKDKCECVGSG------GKERCVCKQKKWAKIIESYIGDIPTLPK-------    62  SN_19_00 (SN_47_01)
sp|B6DCV0|TX501_LYCSI                  KDKENCIPKHHECTSDRHGCCRGSMFKYKCQCVKIVN-AQKEETERCACITPGLHKAAEFVVQLFKKVIA-------    69  SN_19_04
CsTx-33ab_CUPSA                        KDKRNCIPRNQECTIDKRNCCRRGLFKMTCQCMKSND-ESGQPTEKCTCRRPRPIFHLLYKG--LLKG----------   61  SN_19_13
CsTx-9a_CUPSA                          KDDKNCIPKHHECTNDKKNCCKKGLTKMKCKCFTVAD-AKGATSERCACDSSLLQ-KFGFTGLHIIKGLF--------   68  SN_19_13
CsTx-9c_CUPSA                          KDDKNCIPKHHECTNDKKNCCKKGLLKLKCQCFTVAD-EKGTPSERCACGRPLLH-KIAYTGTKMIKGLL--------   68  SN_19_13
                                           :*: ::.:*:  :..**:  : . .*:*            .* * *
```

Percent Identity Matrix - created by Clustal2.1

Nr.	1	2	3	4	5	6
1: tr|A0A059T2C4|A0A059T2C4_CHEPU_C_term	100.00	27.27	30.43	34.43	34.33	40.30
2: tr|A0A059T2C4|A0A059T2C4_CHEPU_N_term	27.27	100.00	45.45	38.00	35.85	35.85
3: sp|B6DCV0|TX501_LYCSI	30.43	45.45	100.00	39.34	41.79	43.28
4: CsTx-33a,b_CUPSA	34.43	38.00	39.34	100.00	43.33	51.67
5: CsTx-9a_CUPSA	34.33	35.85	41.79	43.33	100.00	75.00
6: CsTx-9c_CUPSA	40.30	35.85	43.28	51.67	75.00	100.00

Supplementary Fig S2.16

16. CsTx-35a

CLUSTAL O (1.2.4) multiple sequence alignment of CsTx-35a from *C. salei* (CUPSA) with (putative) neurotoxins from Nephila pilipes (NEPPI), *Cheiracanthium spp* (CHESP), *Alopecosa marikovskyi* (ALOMA), formerly classified as *Geolycosa sp* (GEOA2), and *Dolomedes fimbriatus* (DOLFI)

```
Identifier                          mature peptide sequence                                      family
NEPPI_DN29416_c0_g1_i2_6            SCNVRGEECLKDIECCSAECVCSTNVCKCGTRPKVKTPRSKPQSLAQRLICRYR        54  SN_35_00
CHESP_DN46747_c0_g4_i1_4            SCNFMGEECTKDIECCSIQCLCTTNECRCGRRPKPSQLRKYFSK----RTCGT-        49  SN_35_00
ALOMA_DN55711_c0_g1_i1_5            SCYSRGEECTKDVECCTVQCLCTTNECRCGKRPRPSELRKYFSK----RTCGN-        49  SN_35_00
CsTx-35a_CUPSA                      SCNFKGEECTKDVDCCTMQCLCTTNDCKCGKRPRPSELRKYFTK----RTCGGT        50  SN_35_00
tr|A0A0K1D8H7|A0A0K1D8H7_DOLFI      SCYLKGEECTKDIDCCTMQCLCTTNDCRCGKRPRPSELRKYFTK----RTCGGT        50  SN_35_00
                                    **   **** **::**: :*:*:** *:** **: .  *.   .       *
```

Post-translational removement

Percent Identity Matrix - created by Clustal2.1

Nr.	1	2	3	4	5
1: NEPPI_DN29416_c0_g1_i2_6	100.00	53.06	46.94	46.00	44.00
2: CHESP_DN46747_c0_g4_i1_4	53.06	100.00	79.59	75.51	75.51
3: ALOMA_DN55711_c0_g1_i1_5	46.94	79.59	100.00	81.63	83.67
4: CsTx-35a_CUPSA	46.00	75.51	81.63	100.00	92.00
5: tr|A0A0K1D8H7|A0A0K1D8H7_DOLFI	44.00	75.51	83.67	92.00	100.00

Supplementary Fig S2.17

17. CsTx-40

CLUSTAL O (1.2.4) multiple sequence alignment of CsTx-40 from *C. salei* (CUPSA) with (putative) neurotoxins from *Alopecosa marikovskyi* (ALOMA), formerly classified as *Geolycosa sp* (GEOA2), *Agelenopsis aperta* (AGEAP), *Dolomedes mizhoanus* (DOLMI), *Viridasius fasciatus* (VIRFA), and *Xysticus cristatus* (XYSCR).

```
Identifier                    mature peptide sequence                                                                family
XYSCR_DN38486_c1_g4_i1        --TSLPDGAVCDKNKDDCQCFGKFHGCVCPWFWSS--DQTCHCEWARKGVCISKLSCPNKGEWGPHMRSDEESRSPC        73  SN_42_00
VIRFA_DN51796_c0_g1_i1        RGRSLPPGAECDRAKDNCQCYGKWHKCSCPFW-----SGKCYCEMGTKSTCIFKMSCPNKAEWGLNWRNEETERSPC        72  SN_42_00
CsTx-40_CUPSA                 --KSLPDGEECDGNKDDCQCYGKWHKCSCPYFWPWRS-GKCYCEKGWKHTCITKLSCPNKDEWGLEWRNEEAERSPC        74  SN_42_00
sp|P15969|TOG1A_AGEAP         -AKALPPGSVCDGNESDCKCYGKWHKCRCPWKWHFTGEGPCTCEKGMKHTCITKLHCPNKAEWGLDWRSEESERSPC        76  SN_42_00
tr|S5N3S4|S5N3S4_DOLMI        --KSLPEGAECDGDGSDCQCYGKWHKCGCPFFWKMRG-LKCHCTWGMKHTCITKLSCPNKSEWGLDWRSEESGRSPC        74  SN_42_02
ALOMA_DN64531_c1_g1_i3        ---SLPPGAECDGDKSDCQCYGKWHKCGCPWF---GG-GKCTCQKGYKHTCITKLSCPNKDEWGLDWRSEESERSPC        70  SN_44_00
                                 :** *  **   .:*:*:**:* * **:         * *   .  * .** *: **** ***  . *.:*  ****
```

Percent Identity Matrix - created by Clustal2.1

Nr.	1	2	3	4	5	6
1: CsTx-40_XYSCR	100.00	55.71	61.11	54.79	59.72	61.76
2: CsTx-40_VIRFA	55.71	100.00	76.81	63.38	68.12	73.53
3: CsTx-40_CUPSA	61.11	76.81	100.00	70.27	72.97	80.00
4: sp|P15969|TOG1A_AGEAP	54.79	63.38	70.27	100.00	71.62	80.00
5: tr|S5N3S4|S5N3S4_DOLMI	59.72	68.12	72.97	71.62	100.00	84.29
6: CsTx-40_ALOMA	61.76	73.53	80.00	80.00	84.29	100.00

Supplementary Fig S2.18

18. CsTx-27

CLUSTAL O (1.2.4) multiple sequence alignment of CsTx-27 from *C. salei* (CUPSA) with (putative) neurotoxins from *Alopecosa marikovskyi* (ALOMA), formerly classified as *Geolycosa sp* (GEOA2), and *Lycosa singoriensis* (LYCSI)

```
Identifier                  mature peptide sequence                                               family
CsTx-27_CUPSA               FMLRVGAVCDGNKSDCQCLGKWIKCGCPFLWPMR-SGPCHCTKGRRYTYNKKLSCSNRYLWASEKSR      66  SN_44_00
ALOMA_DN64531_c1_g1_i2_4    MALPPGAECNGHKSDCQCFGAKYKCSCPFLWRFRRNAKCHCKKGWAWTAIKKRSCRNRYQWSD----      63  SN_44_00
sp|B6DD33|TX2M5_LYCSI       MALPPGAVCIGHKSDCQCFGAKYKCSCPFLWRFRRSAKCHCRKGWAWTAIKKRSCHNRYQWSG----      63  SN_44_00
                            : *  ** * *:******:*   :*.**:** :* ..  *** **   :*   ** ** *** *:.
```

Post-translational removement

Percent Identity Matrix - created by Clustal2.1

Nr.	1	2	3
1: CsTx-27_CUPSA	100.00	53.97	58.06
2: ALOMA_DN64531_c1_g1_i2_4	53.97	100.00	90.48
3: sp|B6DD33|TX2M5_LYCSI	58.06	90.48	100.00

Supplementary Fig S2.19

19. CsTx-20

CLUSTAL O (1.2.4) multiple sequence alignment of CsTx-20 from *C. salei* (CUPSA) with (putative) neurotoxins from *Viridasius fasciatus* (VIRFA), the scorpion *Hadrurus spadix* (HADSP), *Parasteatoda tepidariorum* (PARTE), *Nephila pilipes* (NEPPI), *Heteropoda venatoria* (HETVE), *Stegodyphus mimosarum* (STEMI), *Dolomedes sulfureus* (DOLSU), and *Dolomedes fimbriatus* (DOLFI)

```
Identifier                        mature peptide sequence                                                                   family
sp|P25687|MIT1_DENPO              AVITGACERDLQCGKGTCCAVSLWIKSVRVCTPVGTSGEDCHPASHKIPFSGQRMHHTCPCAPNLACVQTSPK---------KFKCLSKS---     81  SN_20_00
tr|A0A1V0FW55|A0A1V0FW55_VIRFA    -----SCFTDADCESDECCISMVF-VR-GFCHTLGKEGDHCIDKPADTEH-GKKNLFGCPCREGYKCIAEVTQ-EGEKTVFKNPTCREGGE--     82  SN_20_00
tr|A0A1W7R966|A0A1W7R966_HADSP    --FGNSCESGKDCPENECCLRRYFSTR---CEKLREEGEFCSPRNENGTE-RAVYMLFCPCESGLQCIPETVEDRNGTTIYPNLKCTSP----     83  SN_20_00
tr|A0A1W7R967|A0A1W7R967_HADSP    ---RDGCESGEDCPQRECCLRRHFTKS---CEMLTEEGEFCSPRNENGTE-RAVYMLFCPCESGLQCIPKTVEDRNGTTIYPNLKCTRQYSED     86  SN_20_00
tr|A0A2L2YRH1|A0A2L2YRH1_PARTE    --AHKDCKPQSDCGEDECCLNYLFFKR-PHCYPRYGAGKRCITLSRYDED-KDQFLVSCPCVKGHECKGEEVE-EDGVSIELS----------     78  SN_20_00
tr|A0A076L065|A0A076L065_NEPPI    ---AQKCTKQSDCEPDECCLDTLFFRH-AFCEPRYKAGQSCPTASLYRAE-KDLYYFACPCVDKYECLGKGSL-EKGVTVIKNAKCIMPTV--     85  SN_20_00
tr|A0A088BPC3|A0A088BPC3_HETVE    ---YTSCTKQSDCEEDECCLDTLFFKR-AYCEKRYAAGSACPTASVYKPE-EDMFYLACPCVPMYECLGKGVTGEDGKTYMKDTKCIMPL---     85  SN_20_02
tr|A0A087V0D4|A0A087V0D4_STEMI    ---ATRCTQQSDCGEDECCLDTLFFKS-AFCEKRYKAGQRCVATSIYKPE-KDMFYFACPCEKMYECLGKGVT-ENGVTFMKNPKCIMPTM--     85  SN_20_00
```

```
sp|B3EWT5|TXC20_CUPSA               ---WNACTKQSDCEEDECCLDNLFFKR-PYCEKRYGAEQRCSAAAVYKED-KDLYYFTCPCVPMYECLGKGSLDENGNTVMKNPKCIMPTL--    86  SN_20_01
tr|A0A0P0DQ99|A0A0P0DQ99_DOLSU      ---WTACTKQSDCEEDECCLDNLFFKR-PYCEKRYGAEQRCSATAIYKEE-KDLYYFTCPCVQMYECLGKGTTDENGNTVMKDPKCIMPTR--    86  SN_20_01
tr|A0A0K1D8H9|A0A0K1D8H9_DOLFI      ---YTACTKQSDCAEDECCLDNLFFKR-PYCEKRYGAGQRCSATAIYKEE-KDLYYLTCPCVAMYECLGKGSTDENGNTIMKNPKCIMPTR--    86  SN_20_01
                                          *    :*    **    :      *       . *                 ***     *               .
```

Percent Identity Matrix - created by Clustal2.1

Nr.		1	2	3	4	5	6	7	8	9	10
1:	tr|A0A1V0FW55|A0A1V0FW55_VIRFA	100.00	30.77	26.25	29.73	31.71	29.63	30.49	31.71	31.71	29.27
2:	tr|A0A1W7R966|A0A1W7R966_HADSP	30.77	100.00	81.71	30.26	29.63	34.15	34.57	35.37	34.15	39.02
3:	tr|A0A1W7R967|A0A1W7R967_HADSP	26.25	81.71	100.00	28.00	28.92	30.12	33.73	30.95	30.95	35.71
4:	tr|A0A2L2YRH1|A0A2L2YRH1_PARTE	29.73	30.26	28.00	100.00	45.45	49.35	50.65	49.35	48.05	50.65
5:	tr|A0A076L065|A0A076L065_NEPPI	31.71	29.63	28.92	45.45	100.00	65.48	68.24	67.06	63.53	63.53
6:	tr|A0A088BPC3|A0A088BPC3_HETVE	29.63	34.15	30.12	49.35	65.48	100.00	72.62	67.06	68.24	69.41
7:	tr|A0A087V0D4|A0A087V0D4_STEMI	30.49	34.57	33.73	50.65	68.24	72.62	100.00	68.24	72.94	74.12
8:	sp|B3EWT5|TXC20_CUPSA	31.71	35.37	30.95	49.35	67.06	67.06	68.24	100.00	89.53	86.05
9:	tr|A0A0P0DQ99|A0A0P0DQ99_DOLSU	31.71	34.15	30.95	48.05	63.53	68.24	72.94	89.53	100.00	90.70
10:	tr|A0A0K1D8H9|A0A0K1D8H9_DOLFI	29.27	39.02	35.71	50.65	63.53	69.41	74.12	86.05	90.70	100.00

Supplementary Fig S2.20

20. CsTx-22a,b,c

CLUSTAL O (1.2.4) multiple sequence alignment of CsTx-22a,b,c from *C. salei* (CUPSA) with (putative) neurotoxins from the tick *Amblyomma cajennense* (AMBCA), *Parasteatoda tepidariorum* (PARTE), *Haplopelma hainanum* (HAPHA), *Grammostola rosea* (GRARO), *Trittame loki* (TRILO), *Phoneutria nigriventer* (PHONI), *Viridasius fasciatus* (VIRFA), and *Dolomedes fimbriatus* (DOLFI)

```
Identifier              mature peptide sequence                                                      family
tr|A0A023FBL2|AMBCA     -ADEGGCSSDSDCSEGQCCVGSE-ERKCADFAGIGQPCSPKNEPH--HKNCPCQPRFTCVPKGEGYNFCQAS   68  SN_32_00
tr|A0A2L2XXI9|PARTE     AVAVEYC-GKSPCGEGQCCTGSSFHRFCGYLAGEGELCEQPNSDEYYTMACPCEAGLTCLD----NNRCERS   67  SN_32_01
sp|D2Y2C0|H14A1_HAPHA   -----ECWSQADCSDGHCCAGSSFSKNCQPYGGDGEQCEPRNKYEVYSTGCPCEENLMCSV----INRCQSA   63  SN_18_00
tr|H7CEL1|H7CEL1_GRARO  -SGQTACSTQEDCPDGSCCAGPSFAKRCRFYGGEMAQCEPPNRFNEYSTGCPCQEGFICSA----INRCQAA   67  SN_18_00
sp|W4VSC2|PE1_TRILO     ---ETYC-GSQLCGEGYCCTGGHFRRQCRPLADEGQQCEKENKYNDYKLGCPCKGGMICSD----IKYCQKL   64  SN_32_01
sp|P83997|TX16_PHONI    -IPGQSC-TNADCGEGQCCTGGSYNRHCQSLSDDGKPCQRPNKYDEYKFGCPCKEGLMCQV----INYCQKK   66  SN_32_02
tr|A0A1V0FWD1|VIRFA     -VQGKYCISTAECGEGMCCTGGSFNKHCQSLSDEGRPCQRPNEYEDYRTGCPCKEGLFCSV----INYCQKA   67  SN_32_02
tr|S5MK94|DOLMI         -TEGKFCSSSAECGEGSCCTGGSFNRHCQSLAENGTPCQQPNKYDHYSTGCPCKEGLVCSA----INYCQKA   67  SN_32_02
CsTx-22b_CUPSA          -IQGDYCSSTSECGEGMCCTGSSFNRHCQRLSENGTPCQRRNDQDYYTYGCPCQEGLICSV----INYCQEA   67  SN_32_02
CsTx-22a_CUPSA          -IQGKFCSSTSDCGEGMCCTGGSFNRHCQRLSENGRPCQRPNDQDYYSTGCPCKEGLICSI----INYCQEA   67  SN_32_02
CsTx-22c_CUPSA          -IQGKFCSSTSDCGEGMCCTGGSFNRHCQSLSENGRPCQRPNDQDYYSTGCPCKEGLICSI----INYCQEA   67  SN_32_02
                              *     * :* **.*    : *   .     *.  *  .     ***:  : *       : *:
                             Loop 3  ----
```

Percent Identity Matrix - created by Clustal2.1

Nr.	1	2	3	4	5	6	7	8	9	10	11

		1	2	3	4	5	6	7	8	9	10	11
1:	tr|A0A023FBL2|AMBCA	100.00	39.68	38.33	35.94	34.43	34.92	32.81	35.94	39.06	37.50	37.50
2:	tr|A0A2L2XXI9|PARTE	39.68	100.00	43.55	36.36	45.31	40.91	45.45	43.94	46.97	43.94	43.94
3:	sp|D2Y2C0|H14A1_HAPHA	38.33	43.55	100.00	60.32	41.94	50.00	53.97	52.38	49.21	49.21	49.21
4:	tr|H7CEL1|H7CEL1_GRARO	35.94	36.36	60.32	100.00	43.75	39.39	43.28	46.27	41.79	46.27	46.27
5:	sp|W4VSC2|PE1_TRILO	34.43	45.31	41.94	43.75	100.00	51.56	54.69	54.69	46.88	48.44	48.44
6:	sp|P83997|TX16_PHONI	34.92	40.91	50.00	39.39	51.56	100.00	69.70	68.18	62.12	66.67	68.18
7:	tr|A0A1V0FWD1|VIRFA	32.81	45.45	53.97	43.28	54.69	69.70	100.00	73.13	70.15	74.63	76.12
8:	tr|S5MK94|DOLMI	35.94	43.94	52.38	46.27	54.69	68.18	73.13	100.00	68.66	76.12	77.61
9:	CsTx-22b_CUPSA	39.06	46.97	49.21	41.79	46.88	62.12	70.15	68.66	100.00	85.07	83.58
10:	CsTx-22a_CUPSA	37.50	43.94	49.21	46.27	48.44	66.67	74.63	76.12	85.07	100.00	98.51
11:	CsTx-22c_CUPSA	37.50	43.94	49.21	46.27	48.44	68.18	76.12	77.61	83.58	98.51	100.00

CLUSTAL O (1.2.4) multiple sequence alignment of CsTx-22a,b,c from *C. salei* (CUPSA) with the prokineticin Bm8f from the toad *Bombina maxima* and MIT1 from the black mamba *Dendroaspis polylepis polylepis*

```
Identifier                  mature peptide sequence                                                                      family
CsTx-22b_CUPSA             -IQGDYCSSTSECGEGMCCTGSSFNR---HCQRLSENGTPCQR-----RNDQDYYTYGCPCQEGLICSVINYCQEA-----      67   SN_32_02
CsTx-22a_CUPSA             -IQGKFCSSTSDCGEGMCCTGGSFNR---HCQRLSENGRPCQR-----PNDQDYYSTGCPCKEGLICSIINYCQEA-----      67   SN_32_02
CsTx-22c_CUPSA             -IQGKFCSSTSDCGEGMCCTGGSFNR---HCQSLSENGRPCQR-----PNDQDYYSTGCPCKEGLICSIINYCQEA-----      67   SN_32_02
sp|Q8JFX8|BM8F_BOMMX       AVITGVCDRDAQCGSGTCCAASAFSRNIRFCVPLGNNGEECHPASHKVPSDGKRLSSLCPCNTGLTCSKSG-EKYQCS---      77   SN_18_00
sp|P25687|MIT1_DENPO       AVITGACERDLQCGKGTCCAVSLWIKSVRVCTPVGTSGEDCHPASHKIPFSGQRMHHTCPCAPNLACVQTSPKKFKCLSKS      81   SN_18_00
                            :    *.   :**.* **: . : :    *  :. .*  *:        . .     ***  .* *    .  :
                                Loop 3  ----
```

Percent Identity Matrix - created by Clustal2.1

Nr.	1	2	3	4	5
1: CsTx-22b_CUPSA	100.00	85.07	83.58	33.33	22.39
2: CsTx-22a_CUPSA	85.07	100.00	98.51	34.85	22.39
3: CsTx-22c_CUPSA	83.58	98.51	100.00	34.85	22.39
4: sp|Q8JFX8|BM8F_BOMMX	33.33	34.85	34.85	100.00	51.95
5: sp|P25687|MIT1_DENPO	22.39	22.39	22.39	51.95	100.00

Supplementary Fig S2.21

21. CsTx-21a,b,c,d,e,f

CLUSTAL O (1.2.4) multiple sequence alignment of CsTx-22a,b,c,d,e,f from *C. salei* (CUPSA) with (putative) neurotoxins from the pseudoscorpion *Synsphyronus apimelus* (Sapi), *Hadronyche infensa* (HADIN), *Parasteatoda tepidariorum* (PARTE), *Trittame loki* (TRILO), *Poecilotheria formosa* (POEFO), *Haplopelma hainanum* (HAPHA), *Stegodyphus mimosarum* (STEMI), *Phoneutria nigriventer* (PHONI), *Viridasius fasciatus* (VIRFA), *Dolomedes fimbriatus* (DOLFI), and *Heteropoda venatoria* (HETVE)

```
Identifier                  mature peptide sequence                                                                family
Sapi_DN97436_c0_g1_i1       ---QTVCQSDYDCGPDACCKGSFYAGRKCRRLGRVGDVCDLPIN---KDRQMYCPCLPGLVCTIPLGDRSASCQEEQR 72  SN_32_00
```

```
tr|A0A1D0BPN2|A0A1D0BPN2_HADIN      ----QTCGGPDDCGEGSCCVG-SFS-RKCLLLSREGASCQPEYQHTGGIYSYACPCENGYICSPINR-----CQKA-- 65  SN_32_01
tr|A0A2L2XXI9|A0A2L2XXI9_PARTE      ----VEYCGKSPCGEGQCCTGSSFH-RFCGYLAGEGELCEQPNS--DEYYTMACPCEAGLTCLDNNR-----CERS-- 64  SN_32_01
sp|W4VSC2|PE1_TRILK                 ----ETYCGSQLCGEGYCCTGGHFR-RQCRPLADEGQQCEKENK--YNDYKLGCPCKGGMICSDIKY-----CQKL-- 64  SN_32_01
tr|A0A1V0FWG9|A0A1V0FWG9_POEFO      ------CGSQADCSEGSCCAGPSFA-KNCRRYGDEGVQCEPWNK--YEEYSTGCPCKENMICSAINR-----CQKA-- 62  SN_18_00
sp|D2Y2E1|H14C1_HAPHA               -----ECWSQADCSDGHCCAGSSFS-KNCRPYGGDGEQCEPRNK--YEVYSTGCPCEENLMCSVINR-----CQSA-- 63  SN_18_00
tr|A0A087TT19|A0A087TT19_STEMI      -----QMPCPRECEEDECCTGGGYN-RHCRKLGGEMEQCQAKNK--YNDYRTACPCQEGYFCSVIGR-----CQKYE- 64  SN_32_01
sp|P83997|TX16_PHONI                QWIPGQSCTNADCGEGQCCTGGSYN-RHCQSLSDDGKPCQRPNK--YDEYKFGCPCKEGLMCQVINY-----CQKK-- 68  SN_32_02
tr|A0A1V0FWD1|A0A1V0FWD1_VIRFA      ----KYCISTAECGEGMCCTGGSFN-KHCQSLSDEGRPCQRPNE--YEDYRTGCPCKEGLFCSVINY-----CQKA-- 64  SN_32_02
tr|A0A0K1D8G2|A0A0K1D8G2_DILFI      ----KYCSSSSECGEGMCCTGGSYN-RHCQRLAEDGTPCQRPNK--YDQYSTGCPCKEGLLCSVINY-----CQKA-- 64  SN_32_02
tr|A0A088BPQ1|A0A088BPQ1_HETVE      ----GQPCAGGECGENECCAGGSYH-RCCRRLGNDGEPCEEPNR--FNSYLNACPCGEGLFCSVINR-----CQKP-- 64  SN_32_01
CsTx-21e_CUPSA                      ---EPVSCGGEYCREGECCAGGSYH-RNCRSYGDPGDICQKPNK--FNEYRTACPCKEGLICSVINR-----CQKV-- 65  SN_32_01
CsTx-21f_CUPSA                      ---EPVSCGGEYCREGECCAGGSYH-RNCRSYGDPGDICQKPNK--FNEYRTACPCKEGLTCSVINR-----CQKV-- 65  SN_32_01
CsTx-21a_CUPSA                      ---EPVSCGDGYCGEGQCCSGTHYN-PHCKFYGDDGDICQRPNK--YNQYKTACPCKEGLTCNVINR-----CQRD-- 65  SN_32_01
CsTx-21c_CUPSA                      ---EPVLCGDGYCGEGQCCSGTHYN-PHCKFYGDDGDICQRPNK--YNQYKTACPCKEGLTCNVINR-----CQRD-- 65  SN_32_01
CsTx-21b_CUPSA                      ---EPISCGDGYCGEGQCCSGTHYN-PHCKFYGDDGDICQRPNN--YNQYKTACPCKEGLTCNVINR-----CQRD-- 65  SN_32_01
CsTx-21d_CUPSA                      ---EPVSCGDGYCGEGQCCSGTHYN-PHCKFYGDDGDICQRPNN--YNQYKTACPCKEGLTCNVINR-----CQRD-- 65  SN_32_01
                                                *  . ** *  :    *   .     *:            ***  .  *          *:
                                         Loop 3  ----
```

Percent Identity Matrix - created by Clustal2.1

Nr.	1	2	3	4	5	6	7	8	9	10	11	12	13	14	15	16	17
1: Sapi_DN97436_c0_g1_i1	100.00	33.87	31.75	30.16	32.79	32.26	31.75	32.81	33.33	36.51	36.51	34.38	34.38	34.38	34.38	34.38	35.94
2: tr|A0A1D0BPN2|A0A1D0BPN2_HADIN	33.87	100.00	42.86	46.03	52.46	45.16	41.94	42.86	47.62	46.03	41.27	44.44	42.86	38.10	38.10	38.10	38.10
3: tr|A0A2L2XXI9|A0A2L2XXI9_PARTP	31.75	42.86	100.00	45.31	37.10	42.86	36.51	42.19	42.19	42.19	45.31	42.19	43.75	43.75	43.75	43.75	43.75
4: sp|W4VSC2|PE1_TRILK	30.16	46.03	45.31	100.00	51.61	41.27	47.62	51.56	51.56	51.56	43.75	50.00	48.44	45.31	45.31	43.75	43.75
5: tr|A0A1V0FWG9|A0A1V0FWG9_9ARAC	32.79	52.46	37.10	51.61	100.00	74.19	43.55	48.39	58.06	53.23	45.16	56.45	54.84	45.16	45.16	43.55	43.55
6: sp|D2Y2E1|H14C1_HAPHA	32.26	45.16	42.86	41.27	74.19	100.00	42.86	46.03	52.38	50.79	46.03	49.21	49.21	44.44	44.44	42.86	42.86
7: tr|A0A087TT19|A0A087TT19_9ARAC	31.75	41.94	36.51	47.62	43.55	42.86	100.00	49.21	53.97	52.38	55.56	53.97	53.97	47.62	47.62	46.03	46.03
8: sp|P83997|TX16_PHONI	32.81	42.86	42.19	51.56	48.39	46.03	49.21	100.00	68.75	70.31	54.69	56.92	56.92	60.00	58.46	58.46	58.46
9: tr|A0A1V0FWD1|A0A1V0FWD1_9ARAC	33.33	47.62	42.19	51.56	58.06	52.38	53.97	68.75	100.00	76.56	50.00	53.12	53.12	51.56	51.56	51.56	51.56
10: tr|A0A0K1D8G2|A0A0K1D8G2_9ARAC	36.51	46.03	42.19	51.56	53.23	50.79	52.38	70.31	76.56	100.00	54.69	53.12	53.12	56.25	56.25	54.69	54.69
11: tr|A0A088BPQ1|A0A088BPQ1_HETVE	36.51	41.27	45.31	43.75	45.16	46.03	55.56	54.69	50.00	54.69	100.00	62.50	62.50	50.00	50.00	50.00	50.00
12: CsTx-21e_CUPSA	34.38	44.44	42.19	50.00	56.45	49.21	53.97	56.92	53.12	53.12	62.50	100.00	98.46	67.69	66.15	64.62	66.15
13: CsTx-21f_CUPSA	34.38	42.86	43.75	48.44	54.84	49.21	53.97	56.92	53.12	53.12	62.50	98.46	100.00	69.23	67.69	66.15	67.69
14: CsTx-21a_CUPSA	34.38	38.10	43.75	45.31	45.16	44.44	47.62	60.00	51.56	56.25	50.00	67.69	69.23	100.00	98.46	96.92	98.46
15: CsTx-21c_CUPSA	34.38	38.10	43.75	45.31	45.16	44.44	47.62	58.46	51.56	56.25	50.00	66.15	67.69	98.46	100.00	95.38	96.92
16: CsTx-21b_CUPSA	34.38	38.10	43.75	43.75	43.55	42.86	46.03	58.46	51.56	54.69	50.00	64.62	66.15	96.92	95.38	100.00	98.46
17: CsTx-21d_CUPSA	35.94	38.10	43.75	43.75	43.55	42.86	46.03	58.46	51.56	54.69	50.00	66.15	67.69	98.46	96.92	98.46	100.00
